# Implementing MR‐PRESSO and GCTA‐GSMR for pleiotropy assessment in Mendelian randomization studies from a practitioner's perspective

**DOI:** 10.1002/gepi.22207

**Published:** 2019-05-02

**Authors:** Jue‐Sheng Ong, Stuart MacGregor

**Affiliations:** ^1^ Statistical Genetics Laboratory, Genetics and Computational Biology Department QIMR Berghofer Medical Research Institute Brisbane Australia

**Keywords:** causal inference, genome‐wide complex trait analysis‐generalized summary mendelian randomization (GCTA‐GSMR), mendelian randomization, mendelian randomization pleiotropy RESidual sum and outlier (MR‐PRESSO), pleiotropy assessment

## Abstract

With the advent of very large scale genome‐wide association studies (GWASs), the promise of Mendelian randomization (MR) has begun to be fulfilled. However, whilst GWASs have provided essential information on the single nucleotide polymorphisms (SNPs) associated with modifiable risk factors needed for MR, the availability of large numbers of SNP instruments raises issues of how best to use this information and how to deal with potential problems such as pleiotropy. Here we provide commentary on some of the recent advances in the MR analysis, including an overview of the different genetic architectures that are being uncovered for a variety of modifiable risk factors and how users ought to take that into consideration when designing MR studies.

Mendelian Randomization (MR) is an approach which uses genetic data to infer if a risk factor is causally related to an outcome. It utilizes the random assortment of variants at meiosis to mimic a pseudo‐randomised controlled trial. Essentially, if variants associated with a risk factor are also associated with the outcome of interest then, subject to some assumptions, a causal relationship may be inferred (Lawlor, 2016). Power is a major rate‐limiting step in MR. Many early MR studies used a one‐sample approach where the SNP‐exposure and SNP‐outcome associations were determined within a single data set. However, such an approach fails to capitalize on the power now available via consortia scale genome‐wide association studies (GWASs), where more SNP instruments can be identified in the SNP‐exposure step and/or where the size of the SNP‐outcome data set is increased. It is hence now common to employ two sample approaches, which uses the largest possible datasets, with the inverse variance weighted (IVW) method used to combine estimates across SNPs (Pierce & Burgess, 2013). However, the IVW approach can yield biased estimates in the presence of horizontal pleiotropy. Two classes of approach try to address this; first approaches on the basis of for example, the median (Bowden, Davey Smith, Haycock, & Burgess, 2016) or mode (Hartwig, Davey Smith, & Bowden, 2017) can provide more robust estimates. Second, approaches on the basis of Egger regression can be used, resulting in valid inference in a broader set of scenarios (InSIDE assumption (Bowden, Davey Smith, & Burgess, 2015)). Whilst these approaches complement IVW estimates, allowing better triangulation of evidence on causality, these estimators are less efficient, reducing power (wider confidence intervals on the causal estimates).

An overview of these approaches has been previously described (Burgess, Timpson, Ebrahim, & Davey Smith, 2015; Zheng et al., 2017), although this is an active area of research, with new ongoing methods development. Whilst a fully updated review of the literature may be seen as premature, two high profile methodological approaches were published recently, which have the potential to address the pleiotropy issue more reliably. Hence, in this article, we focus on two approaches: (a) the Genome‐wide Complex Trait Analysis‐Generalized Summary Mendelian Randomization (GCTA‐GSMR; Zhu et al., 2018) and (b) Mendelian Randomization Pleiotropy RESidual Sum and Outlier (MR‐PRESSO; Verbanck, Chen, Neale, & Do, 2018) and provide some perspective on their utility from a MR practitioner's point of view.

The GCTA‐GSMR framework developed by Zhu et al. (2018) is a generalized model to draw MR causal inference between any modifiable exposure and outcome of interest, expanded from its previous SMR framework, which was originally developed to evaluate causality between the gene expression and disease outcomes. GSMR builds on previous approaches for modeling multiple correlated SNPs in MR (Burgess et al., 2015), by estimating the Linkage Disequilibrium (LD) between SNPs from a reference sample ‐ this avoids the power loss inherent in only using uncorrelated SNPs. The GCTA‐GSMR model borrows the GCTA‐SMR heterogeneity in dependent instrument (HEIDI) test (Zhu et al., 2016) for assessing heterogeneity in the causal estimates across instruments; the test removes outliers, which may be associated with confounding factors. In addition, GCTA‐GSMR models the error in the SNP‐exposure estimate, a term that was left out in conventional 2‐sample MR models as it was assumed to be negligible in the delta‐approximation when the F‐statistic for the SNP‐exposure association is large (Stephen Burgess, Butterworth, & Thompson, 2013). The method also implemented a multivariate MR framework to investigate mediation and marginal contribution(s) of multiple risk factors on disease outcomes. The software is easy to apply, requiring only SNP‐exposure and SNP‐outcome genetic association estimates and a LD‐matrix to account for correlation between SNP‐instruments. On the other hand, MR‐PRESSO by Verbanck and colleagues (Verbanck et al., 2018) assesses pleiotropy from a different viewpoint. MR‐PRESSO adopts a “leave‐one‐out” approach to evaluate whether a specific SNP‐instrument is driving the difference in computed residual sum of squares (RSS) against simulated expectations. Briefly, the model incorporates three stages to examine the extent of horizontal pleiotropy. First, a global test is conducted to test whether the total RSS (computed by excluding one SNP each turn) is consistent with that expected by chance. The second stage uses the RSS of individual SNP‐instruments to identify outliers. The third stage employs a distortion test to determine the extent to which outliers change the MR causal estimates. Because simulation is used to derive *p* values, the computational requirements are not trivial. Required user‐input is similar to GCTA‐GSMR, although as the approach does not, however, handle correlated SNPs, there is no requirement for a LD‐matrix; instead, SNPs must be pre‐screened for LD.

Although the MR‐PRESSO and GSMR HEIDI approaches tackle pleiotropy within a different framework, they are conceptually similar. Both assume that most SNPs are not strongly affected by horizontal pleiotropy and attempt to control SNP‐heterogeneity by removing SNP‐outliers. In conventional 2‐sample MR techniques, heterogeneity of the causal estimates derived from SNPs is often quantified by the Cochran Q test statistics (Bowden et al., 2018). For MR‐PRESSO and GSMR, the methodological differences mainly come from the choice of formulation of the test‐statistics to quantify statistical heterogeneity and reliance of parametric/non‐parametric solutions (see Table 1).

**Table 1:** Comparison of SNP‐Heterogeneity tests across MR‐PRESSO, gSMR, and classical 2‐sample MR methods

Method	Formulation of heterogeneity test	Test statistics	Description
GCTA‐GSMR HEIDI	$d_{i}^{2}\;={\left(\beta_{i}-\beta_{\text{best}}\right)}^{2}$	The test statistic $T_{i}$ converges to $\chi^{2}\left(\mathrm{df}\;=1\right)$	Computes SNP‐level heterogeneity only, and uses the HEIDI test to discard outliers (e.g. $T_{i}$ *p* value < 0.01). However, theoretically possible to implement a global test.
	$T_{i}\;=d_{i}^{2}/\mathrm{var}\left(d_{i}\right)$		
MR‐PRESSO	$\sum_{i=1}^{k}{\left(\beta_{\mathrm{zy}\left(i\right)}-\beta_{\mathrm{zx}\left(i\right)}\beta_{-i}\right)}^{2}$	Empirical	Relies on bootstrap to generate empirical distribution for the causal estimates. The main difference is that it uses a “leave‐one‐out” approach to obtain unbiased RSS values. Evaluates both SNP‐level and global heterogeneity.
	Note that this can also be rewritten as		
	$\sum_{i=1}^{k}{h_{i}\left(\beta_{i}-\beta_{-i}\right)}^{2}$		
	Where $h_{i}\;={\left(\beta_{\mathrm{zx}\left(i\right)}\right)}^{2}$		
Mode, median, inverse variance weighted models	Cochran Q test:	Convergence to $\chi^{2}\left(\mathrm{df}\;=k\;-1\right)$	Estimates global heterogeneity
	$\sum_{i=1}^{k}{w_{i}\left(\beta_{i}-\beta_{\text{IVW}}\right)}^{2}$		
	$w_{i}\;\approx \frac{\beta_{\mathrm{zx}\left(i\right)}^{2}}{\mathrm{var}\left(\beta_{\mathrm{zy}\left(i\right)}\right)}$ (1st order term)		
Modified MR‐Egger	Cochran Q' test:	Convergence to $\chi^{2}\left(\mathrm{df}\;=k\;-2\right)$	Fits an additional intercept term before adjusting for directional pleiotropy. Also models global heterogeneity
	$\sum_{i=1}^{k}{w_{i}\left(\beta_{i}-\beta_{\text{IVW}}+\beta_{0}\right)}^{2}$		

Abbreviations: GSMR, generalized summary mendelian randomization; HEIDI, heterogeneity in dependent instrument; MR‐PRESSO, mendelian randomization pleiotropy residual sum and outlier.

$\beta_{\mathrm{zx}}$ and $\beta_{\mathrm{zy}}$ refer to the SNP‐exposure and SNP‐outcome association estimate.

$\beta_{i}$ refers to the wald‐type estimator for SNP i, given by $\beta_{i}\;=\beta_{\mathrm{zy}(i)}\;/\beta_{\mathrm{zx}(i)}$.

$\beta_{\mathrm{best}}$ is the GSMR causal estimate for the SNP at top 25‐th percentile of log(*p* value) on the SNP‐exposure association. The reason for not using the SNP with the highest log(*p* value) is to avoid potential SNP‐pleiotropy generating a bias on the test statistics.

$\beta_{\text{IVW}}$ is the inverse‐variance weighted estimate for all SNP instruments.

$\beta_{-j}$ refers to the IVW estimate for all SNP instruments excluding SNP j. Finally, $\beta_{0}$ is the MR‐Egger intercept of the regression.

Each model in the above also assumes that β values follow Gaussian distributions. In other 2‐sample MR models, heterogeneity is often quantified via the Cochran Q test statistics (or Q’ for modified MR Egger).

Despite MR‐PRESSO and GSMR being promising additions to the MR sensitivity toolbox, there are some important limitations. As previously mentioned, the MR‐PRESSO model does not incorporate LD (although theoretically it could be implemented using a multivariate normal simulation framework), which may mean the variance explained in the modifiable risk factor is lower than that attainable when correlated SNPs are included. Although GCTA‐GSMR does allow correlated SNPs to be included, the model relies on the LD reference panel used to inform LD between SNP instruments being reflective of the target sample (Vilhjálmsson et al., 2015) in a 2‐sample MR design. This is unlikely to be an issue for quantitative outcomes but may impact findings for disease outcomes if the LD pattern is substantially different between cases and controls. MR‐PRESSO applies a global distortion test, to evaluate whether the removal of the potentially pleiotropic instrument makes a meaningful difference to the overall causal estimate, whereas GCTA‐GSMR filters SNP‐outlier one at a time and does not apply a global test. Because of its reliance on simulation, the MR‐PRESSO runtime varies. In our test example using publicly available GWAS data (Table 3), 10,000 simulation replicates were sufficient (runtime about 6 minutes). The GCTA‐GSMR runtime was faster although in practice runtime is not a major issue for either method. In common with other MR sensitivity models (Zheng et al., 2017; median, mode, MR Egger), although both approaches can theoretically be applied to a relatively small number of SNP instruments (>5 say), their power to identify outliers is likely to be limited in such scenarios.

Before we further evaluate the feasibility of these MR approaches in practice, it is useful to give some thought to the likely genetic architecture of the exposure of interest. To examine how practical these recently developed models are in terms of modeling pleiotropy, let us first consider three illustrative scenarios for the genetic architecture of the modifiable risk factor of interest (Figure 1) modeled after real traits (coffee consumption; Coffee & Caffeine Genetics Consortium et al., 2015), alcohol intake (Liu et al., 2019), and body mass index (Locke et al., 2015). Power is a key issue in MR and power is directly related to the variance explained by the chosen SNP instruments (Brion, Shakhbazov, & Visscher, 2013). Hence a key consideration is the increase in cumulative *r* ^2^ as increasingly weaker instruments are added (Figure 2). With the total instrument *r* ^2^ calculated, the statistical power for the MR analysis can then be easily estimated using the online MR power calculator, mRnd (http://cnsgenomics.com/shiny/mRnd/) web interface (Brion et al., 2013).

**Figure 1:**
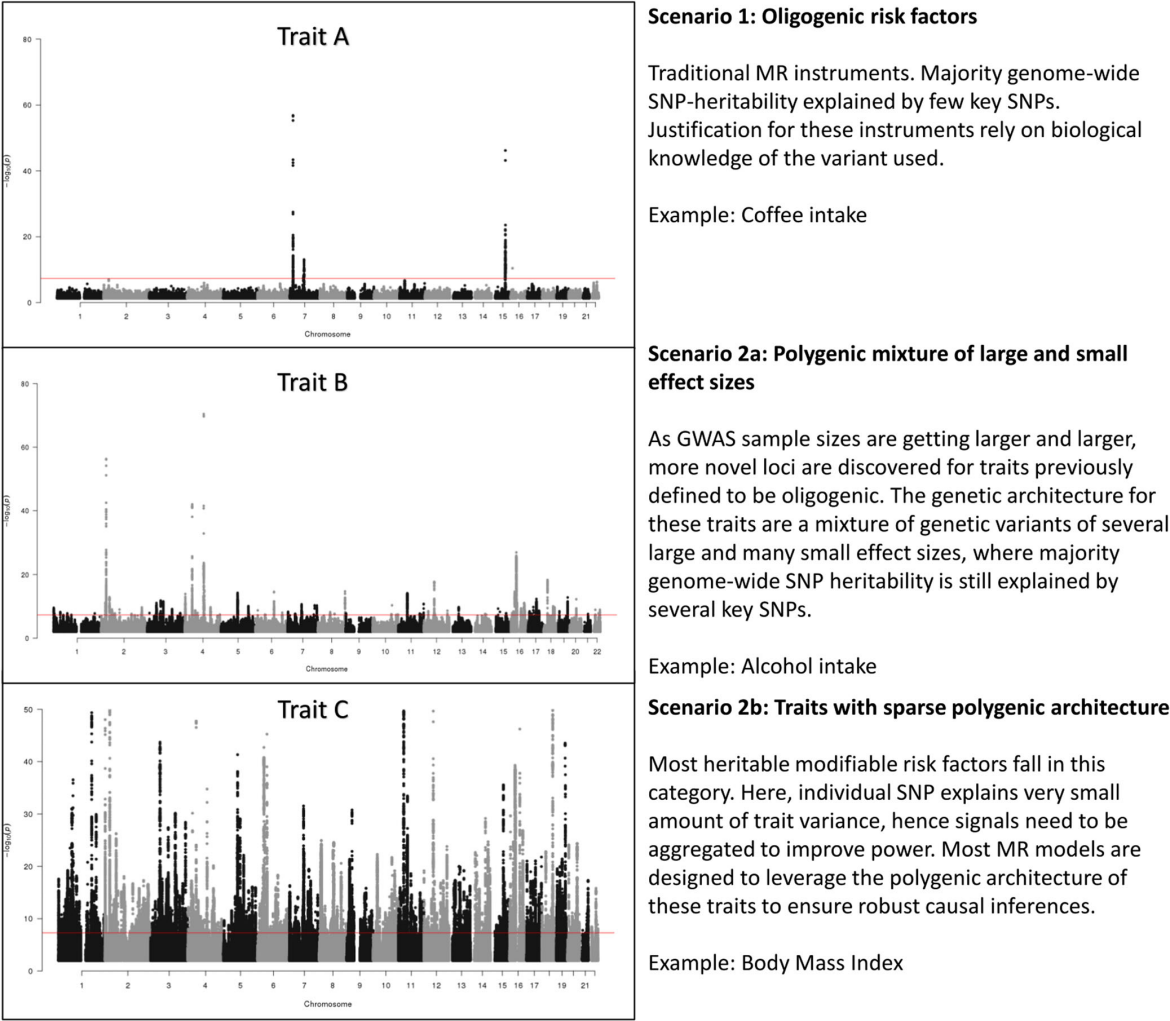
Illustrative scenarios for the genetic architecture of modifiable risk factors. The figure above shows the Manhattan plots (left panel) illustrating the different type of genetic architecture for modifiable risk factors used in MR studies. The red line (at y = log10(5e‐8)) indicates the genome‐wide significance (GW) threshold, where variants with a ‐log10(*p* value) above the line are deemed to be genome‐wide significant. As genome‐wide significant SNPs have F‐statistics > 30, they can be used as viable instruments given the other MR‐assumptions hold. The GWAS for trait A was modeled after coffee consumption; trait B modeled after Alcohol intake; trait C modeled after BMI. Note that the plots above are illustrative and do not represent the current state of knowledge for these traits. GWASs, genome‐wide association studies; SNP, single nucleotide polymorphism

**Figure 2:**
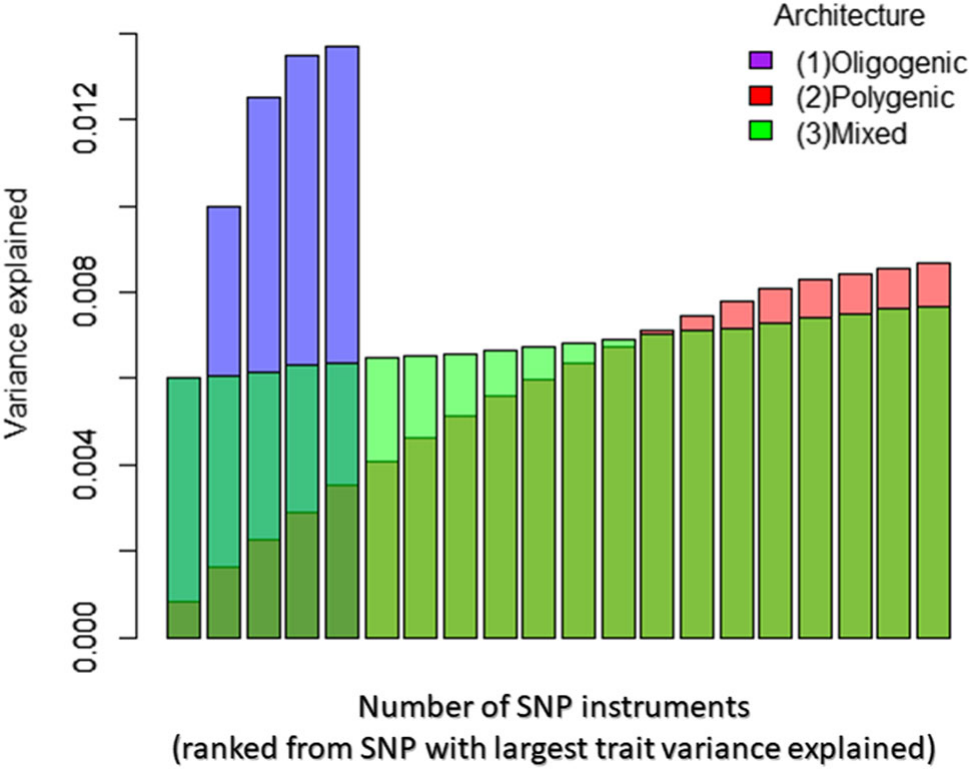
Distribution of cumulative SNP variance explained based on different forms of polygenicity in genetic architecture. The x‐axis represents the cumulative variance explained by SNPs (commonly denoted as r^2) for the underlying trait of interest ‐ an important indicator of power for MR analyses. While the y‐axis refers to the number of instruments starting from the SNP with the largest r^2 on the underlying trait. The mixed form is analogous to Scenario 2a in the main text. The change in cumulative variance explained by instruments can be used to evaluate whether there is any marginal benefit (on power) for including more SNP instruments. SNP, single nucleotide polymorphism

For modifiable risk factors from scenario 1 (Figure 1, top panel), power will be adequate with only a few SNPs; most statistical pleiotropy evaluation methods (including GCTA‐GSMR and MR‐PRESSO) will not be effective in such situations ‐ instead, it is usual to include the few SNP instruments on the basis of biological grounds. In addition, Phenome‐wide Association Studies (Hebbring, 2014; PheWAS) can be conducted to evaluate whether the SNPs affect putative confounders of the exposure‐outcome relationship.

On the basis of scenario 2a (Figure 1, middle panel), if the confidence intervals on the causal odds ratios are sufficiently narrow with just a few SNPs of large effect, then theoretically, the MR analysis can proceed as per scenario 1. However, if additional polygenes are available then these can be used alongside the genes of large effect in a GCTA‐GSMR or MR‐PRESSO analysis ‐ this will provide a statistical evaluation of whether pleiotropy will potentially bias causal inference. This statistical approach may be used to supplement the information on the biological function of specific SNP instruments, where this data is available for the risk factor of interest.

In scenario 2b (Figure 1, bottom panel), power is very unlikely to be sufficient with just a few top SNPs and pleiotropy evaluation for a large number of required polygenes will again be critical. With a large number of SNPs, incorporating biological/functional information for each SNP is unlikely to be tractable and the statistical approaches to identify and remove outliers in GSMR and MR‐PRESSO will be useful. In the flowchart (Figure 3), depending on the anticipated genetic architecture of the modifiable risk factor we provide guidance on the preferred MR approach. An overview of the broad selection of modifiable risk factors commonly used in MR studies is given in Table 2 where MR analyses involving a low number of variants remain prevalent in the literature. Furthermore, Table 2 also shows no clear relationship between total variance explained by instruments and the number of instruments, hence the need to evaluate the genetic architecture for our trait of interest before deciding a sensible approach (Table 3).

**Figure 3:**
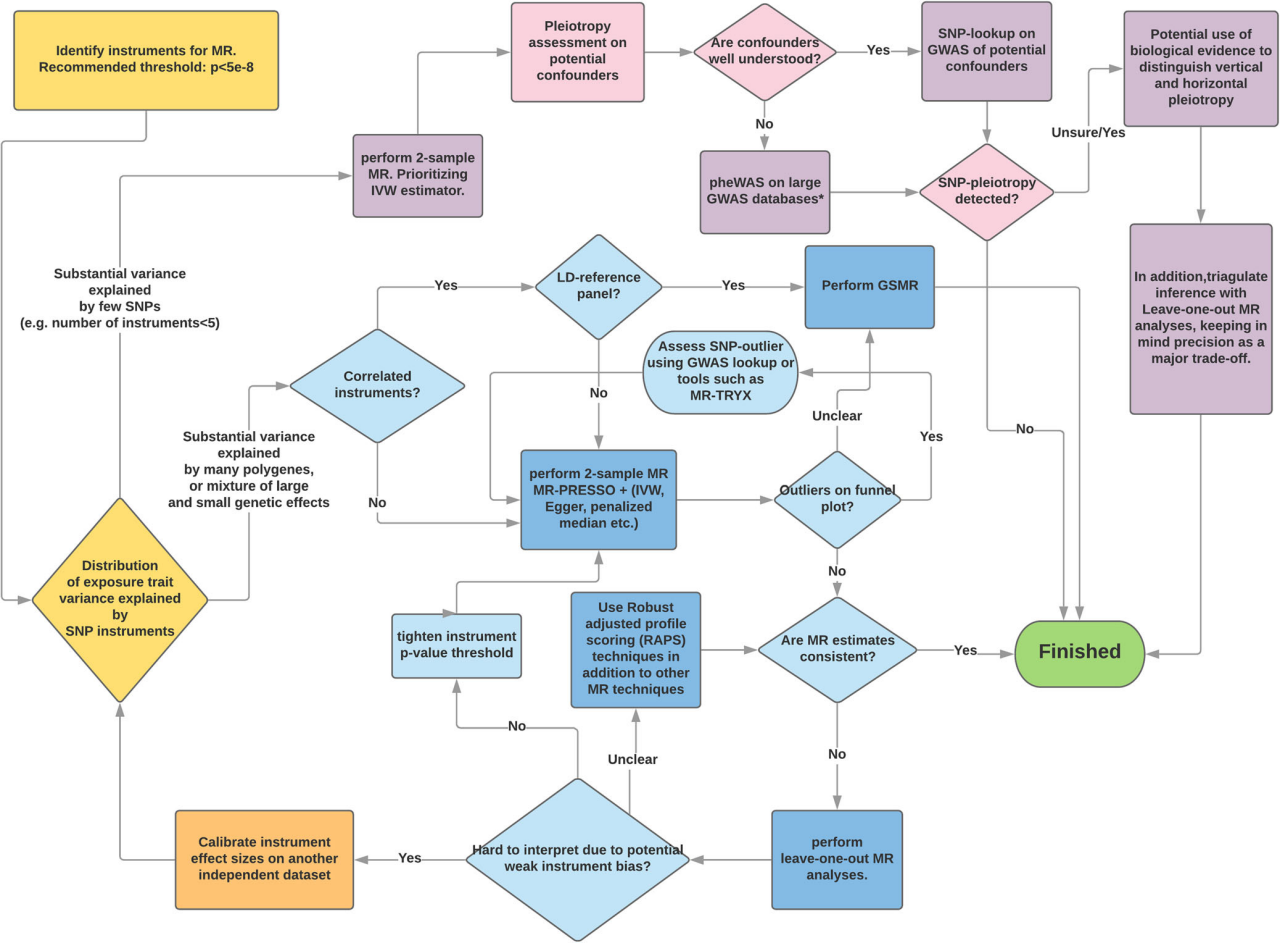
Flowchart outlining approaches for performing two‐sample Mendelian randomization studies. The flowchart outlines some recommended steps to perform MR sensitivity analyses on the basis of the genetic architecture of the modifiable risk factors (Scenario 1, 2a and 2b). The path highlighted in purple refers to techniques commonly applied for traits in Scenario 1, whereas those highlighted in blue are for traits with a more polygenic architecture. In Scenario 1, SNPs that are (or are from genes) potentially associated with other confounding risk factors should first be removed before the main analysis. For Scenario 2, evidence of SNP‐pleiotropy can be identified via outliers on the MR funnel plot. The main difference between two paths is that methods in Scenario 1 rely on biological knowledge of instruments to evaluate pleiotropy whereas more statistical approaches were utilized in Scenario 2. The MR‐TRYX software can be found here: https://github.com/explodecomputer/tryx. SNP, single nucleotide polymorphism

**Table 2:** Selection of commonly used modifiable risk factors in published MR studies

Modifiable risk factor	Number of instruments	Approximate instrument *r* ^2^	PubMed ID
Alcohol intake (European)	1	1%	28645180; 29212772; 25503943
Alcohol intake (Asian)	1	3%	27575649
Age at menarche	375	7%	28436984
Bitter taste liking	1	43%	23900446
Body mass index	73–97	1.4–2.7%	29232439; 27401727; 27427428
Coffee consumption	5	0.60%	29760501
C‐reactive protein	4	2%	20056955
Calcium	1	1%	28742912
Dairy intake	1	1%	28302601; 29071490
Education attainment	162	1.80%	28855160
Fasting glucose	37	5%	28954281
Fasting Insulin	17	1%	28954281
H.pylori susceptibility	2	1%	29089580
Height	>2000	13%	29581483
High‐density lipoprotein (HDL)	63	14%	28594918
Hydroxyvitamin‐D	4	3%	27594614; 29089348; 26305103
Low‐density lipoprotein (LDL)	50	15%	28594918
Plasma vitamin C	1	1%	29939348
Plasma urate	1	2%	28428355
Polyunsaturated fatty acids (multiple)	2–5	8–30%	29473154; 27490808
Serum iron level	5	4%	28186534
Smoking heaviness	1	1%	29509885
Triglyceride	45	12%	28594918
Tobacco consumption	1	1%	29688528
Total cholesterol	65	15%	28594918
Vitamin B12	3–11	3–6%	22199995; 29249824
Waist‐to‐hip ratio (both sexes)	47	1.40%	27550749

The table above represents a selection of some of the risk factors considered in MR studies to date. Note that this list is not a complete representation of all the modifiable traits in the MR literature, but merely to show that traits that MR studies with few instruments remain relevant in the field. Selection of studies are on the basis of the criteria that (a) variance explained by instruments (r^2) are reported and (b) total sample size in the outcome set. r^2 are approximated on the basis of sample size and reported F‐statistics if r^2 is not available from previously cited GWASs or the original article itself.

**Table 3:** Comparison of MR estimates for LDL‐cholesterol on coronary artery disease between GCTA‐GSMR and MR‐PRESSO

Method	Settings	Raw estimate			Outlier adjusted				Runtime, sec	Additional comments
		Causal estimate	SE	*p* value	Causal estimate	SE	*p* value	SNPs filtered		
GCTA‐GSMR	LD‐matrix precomputed	NA	NA	NA	0.432	0.022	4.15E‐85	9	2.5	Runtime was on the basis of the analysis portion only. The computation of the LD‐matrix needs to be done via GCTA separately.
MR‐PRESSO	Nb = 1000, outlier *p* val = 0.05	0.402	0.071	5.02E‐08	0.462	0.027	5.70E‐36	48	39.1	Outlier test unstable with only 1000 simulations to compute the null distribution (i.e. cannot obtain pval of outlier < 0.188).
	Nb = 10,000, outlier *p* val = 0.05	0.402	0.071	5.02E‐08	0.408	0.028	2.56E‐30	45	375.2	
	Nb = 50,000, outlier *p* val = 0.05	0.402	0.071	5.02E‐08	0.408	0.028	2.56E‐30	45	1871.2	

Abbreviations: GCTA‐GSMR, genome‐wide complex trait analysis‐generalized summary mendelian randomization; MR‐PRESSO, mendelian randomization pleiotropy residual sum and outlier; SNP, single nucleotide polymorphism.

Causal estimate refers to the estimated effect size (log(OR) on coronary artery disease (CAD) risk per standard deviation increase in genetically predicted LDL‐cholesterol (LDL‐c). SE refers to the respective standard errors of the causal estimate. Nb denote the number of simulation replicates required to generate the null distribution used in the MR‐PRESSO outlier tests. The data for these traits were extracted from publicly available GWAS summary statistics (LDL‐c from http://csg.sph.umich.edu/willer/public/lipids2013/; CAD from http://www.cardiogramplusc4d.org/data‐downloads/).

The MR flowchart (Figure 3) provide potential guidelines on how to utilize various sensitivity analyses at different stages of the MR analysis. Although we attempt to streamline the process for display in the chart, in practice every step requires critical consideration. First, plotting the cumulative *r* ^2^ (Figure 2) can help breakdown the distribution of variance explained by instruments to evaluate whether sufficient variance can be captured by several SNPs to allow a well‐powered MR. The choice and proposed method for pleiotropy assessment will then depend on whether substantial variance can be captured by only a few SNPs. Although approaches, such as GCTA‐GSMR and MR‐PRESSO offer a statistical approach for dealing with outliers, in some scenarios biological information is available and should be used sensibly (e.g. if one of the SNP instruments explains a high proportion of variance and that SNP has strong pleiotropic effects on putative confounders then it may make sense to drop the SNP before outlier screening in e.g. MR‐PRESSO). Where applicable, bidirectional MR (Davey Smith & Hemani, 2014) can be conducted to clarify horizontal pleiotropy from mediation/vertical pleiotropy. Note that statistical methods (for 2‐sample MR) available in the literature are not limited to those described in Figure 3.

It is becoming clear that many modifiable risk factors of interest in causal inference studies have a polygenic architecture. Although power remains a rate‐limiting step in some applications, with the advent of very large bio‐bank scale studies, there are scenarios where power is reasonable provided one is willing to use large numbers of SNPs as MR instruments. In such situations, the pleiotropy assessments provided by tools, such as MR‐PRESSO and GSMR will be invaluable in enabling effective and robust causal inference using MR.

## CONFLICT OF INTERESTS

The authors declare that there are no conflict of interests.
